# Comparative Transcriptomic and Molecular Pathway Analyses of HL-CZ Human Pro-Monocytic Cells Expressing SARS-CoV-2 Spike S1, S2, NP, NSP15 and NSP16 Genes

**DOI:** 10.3390/microorganisms9061193

**Published:** 2021-05-31

**Authors:** Anshika Sharma, Joe W. Ong, Mun Fai Loke, Eng Guan Chua, Joseph J. Lee, Hyung Won Choi, Yee Joo Tan, Sunil K. Lal, Vincent T. Chow

**Affiliations:** 1 Infectious Diseases Translational Research Program, Department of Microbiology and Immunology, Yong Loo Lin School of Medicine, National University of Singapore, Singapore 117545, Singapore; Anshika.Sharma@monash.edu (A.S.); joe.ong@outlook.com (J.W.O.); lmunfai@gmail.com (M.F.L.); mictyj@nus.edu.sg (Y.J.T.); 2 School of Science, Tropical Medicine and Biology Platform, Monash University Malaysia, Subang Jaya 47500, Selangor, Malaysia; sunil.lal@monash.edu; 3 Marshall Centre for Infectious Diseases Research and Training, University of Western Australia, Perth, WA 6009, Australia; eng.chua@uwa.edu.au; 4 Department of Medicine, Yong Loo Lin School of Medicine, National University of Singapore, Singapore 119228, Singapore; joseph.lee@u.nus.edu (J.J.L.); hyung_won_choi@nus.edu.sg (H.W.C.); 5 Institute of Molecular and Cell Biology, Agency for Science, Technology and Research, Singapore 138673, Singapore

**Keywords:** SARS-CoV-2, RNA-sequencing, Spike S1, Spike S2, Nucleocapsid (NP), endoribonuclease (NSP15), Methyltransferase (NSP16)

## Abstract

The ongoing COVID-19 pandemic is a clear and present threat to global public health. Research into how the causative SARS-CoV-2 virus together with its individual constituent genes and proteins interact with target host cells can facilitate the development of improved strategies to manage the acute and long-term complications of COVID-19. In this study, to better understand the biological roles of critical SARS-CoV-2 proteins, we determined and compared the host transcriptomic responses of the HL-CZ human pro-monocytic cell line upon transfection with key viral genes encoding the spike S1 subunit, S2 subunit, nucleocapsid protein (NP), NSP15 (endoribonuclease), and NSP16 (2′-O-ribose-methyltransferase). RNA sequencing followed by gene set enrichment analysis and other bioinformatics tools revealed that host genes associated with topologically incorrect protein, virus receptor activity, heat shock protein binding, endoplasmic reticulum stress, antigen processing and presentation were up-regulated in the presence of viral spike S1 expression. With spike S2 expression, pro-monocytic genes associated with the interferon-gamma-mediated signaling pathway, regulation of phosphatidylinositol 3-kinase activity, adipocytokine signaling pathway, and insulin signaling pathway were down-regulated, whereas those associated with cytokine-mediated signaling were up-regulated. The expression of NSP15 induced the up-regulation of genes associated with neutrophil degranulation, neutrophil-mediated immunity, oxidative phosphorylation, prion disease, and pathways of neurodegeneration. The expression of NSP16 resulted in the down-regulation of genes associated with S-adenosylmethionine-dependent methyltransferase activity. The expression of NP down-regulated genes associated with positive regulation of neurogenesis, nervous system development, and heart development. Taken together, the complex transcriptomic alterations arising from these viral-host gene interactions offer useful insights into host genes and their pathways that potentially contribute to SARS-CoV-2 pathogenesis.

## 1. Introduction

On December 31, 2019, the World Health Organization (WHO) China Country Office was informed of cases of pneumonia of unknown etiology detected in Wuhan in Hubei province [1]. The WHO characterized the outbreak as a Public Health Emergency of International Concern in January 2020, and a pandemic was subsequently declared in March 2020 [2]. On February 11, 2020, the WHO named the disease “coronavirus disease 2019” (COVID-19), with the etiologic virus being designated as severe acute respiratory syndrome coronavirus 2 (SARS-CoV-2) [3]. SARS-CoV-2 is thought to have emerged as a spillover of an animal coronavirus that subsequently adapted and acquired the capacity for human-to-human transmission, and then rapidly spread in the human population to cause the fifth pandemic since the 1918 influenza pandemic [4]. As of 15 May 2021, more than 162 million COVID-19 cases have been confirmed, resulting in over 3.3 million deaths [5].

SARS-CoV-2 is a positive-sense, single-stranded RNA virus with a genome of ~29 kb that is organized into 11 open reading frames (ORFs) [6]. The first ORF, which comprises two overlapping ORFs (ORF1a and ORF1b), constitutes ~70% of the viral genome. These ORFs encode two polypeptides that are processed into 16 non-structural proteins (NSP1-16) [7]. The remaining ORFs encode structural proteins, i.e., the spike surface glycoprotein (S), envelope (E), membrane (M), nucleocapsid protein (NP), and other accessory proteins, similar to the older SARS coronavirus or SARS-CoV [8,9]. The S protein plays a key role in host cell entry, and consists of two subunits (S1 and S2). The S1 subunit harbors a receptor-binding domain that recognizes and binds to the host receptor angiotensin-converting enzyme 2 (ACE2) to mediate cell entry. The S2 subunit mediates viral cell membrane fusion by forming a six-helical bundle via the two-heptad repeat domain [10]. In the native state, the S protein exists as an inactive precursor. During viral infection, when the S protein binds to the receptor, transmembrane protease serine 2 (TMPRSS2), a type 2 TM serine protease located on the host cell membrane, activates the S protein by cleaving it into S1 and S2 subunits [11]. The N protein is crucial for incorporating genomic RNA into progeny viral particles—N protein recruitment to replication-transcription complexes (RTCs) is an essential step in the viral life cycle [12]. NSP15 of SARS coronavirus has been biochemically characterized as an endoribonuclease (EN) that cleaves viral RNA at uridylates at the 3′-position to form a 2′-3′ cyclic phosphodiester product to prevent the host immune-sensing system from detecting the virus. NSP15 mediates the evasion of RNA sensors, and limits apoptosis in macrophages [13].

NSP16 acts as a 2′-O-ribose-methyltransferase (ME) to methylate the 2′-hydroxy group of adenine using S-adenosylmethionine as the methyl source. The viral RNA has a 5′-cap, which protects it from mRNA degradation by 5′-exoribonucleases, promotes mRNA translation, and prevents the viral RNA from being recognized by innate immune mechanisms. This RNA cap is an N7-methylated guanine nucleotide connected through a 5′-5′ triphosphate bridge to the first transcribed nucleotide (adenine) [14].

Interactions between host and virus involve an intricate interplay between molecular pathways initiated by the mechanisms triggered by the virus to successfully infect the cell and by the host in response to the infection. In order to achieve a clearer understanding of the roles of five key SARS-CoV-2 proteins (namely spike S1, S2, NP, NSP15, and NSP16) in the host monocytic responses and pathophysiology of COVID-19, we employed RNA sequencing (RNA-Seq) to investigate gene expression changes in the HL-CZ pro-monocytic cells following transfections with the viral genes. HL-CZ is a cell line of monocytic lineage that possesses CD15, CD34 and CD11b markers, and is permissive for the propagation of SARS-CoV [15]. The severity and mortality of COVID-19 patients are associated with substantial mononuclear cell infiltration in their lungs and other organs, along with elevated levels of circulating cytokines and chemokines including IL-6, TNF and CXCL10. Bronchoalveolar lavage fluid from severe COVID-19 patients contain abundant mononuclear phagocytes (MNPs) and inflammatory monocyte-derived macrophages. In patients who succumbed to COVID-19, post-mortem analyses reveal the presence of SARS-CoV-2 viral particles and NP antigen within lymph node and splenic macrophages. It is thought that aberrant MNP activation or macrophage activation syndrome culminates in the cytokine release syndrome or “cytokine storm” which contributes to hyperinflammation and coagulopathy associated with severity and death in COVID-19 patients [16]. Analyses of the complex downstream transcriptomic alterations that arise from these viral-host gene interactions offer useful insights into how host genes and their pathways are modulated by these viral proteins, especially in monocytes, thereby contributing to SARS-CoV-2 pathogenesis.

## 2. Materials and Methods

### 2.1. Expression Plasmids and Vector Controls

The SARS-CoV-2 expression plasmid constructs were pCMV3-S1 (VG40591-UT) for spike S1, pCMV3-S2-long (VG40590-UT) for spike S2, pCMV3-NP-Flag (VG40588-CF) for nucleocapsid, pCMV3-EN (VG40597-UT) for NSP15 endoribonuclease, and pCMV3-ME (VG40598-UT) for NSP16 methyltransferase. The pCMV3-untagged negative control vector (CV011), and pCMV3-C-FLAG negative control vector (CV012) served as reference controls. The plasmids were purchased from Sino Biological (Beijing, China), and plasmid preparations were subjected to Sanger sequencing using T7 primer (5’–TAATACGACTCACTATAGGG–3’) to ensure the absence of undesired mutations prior to cell transfection.

### 2.2. HL-CZ Cell Culture

HL-CZ is a suspension cell line derived from human pro-monocytic leukemia, and was cultured as described previously [15]. The HL-CZ cells also express ACE2, and higher levels of the FcγRII receptor [17].

### 2.3. Antibodies

The primary and secondary antibodies (Sino Biological, Beijing, China) employed for Western blot and immunofluorescence analyses were rabbit polyclonal antibody against SARS-CoV-2 spike S1 (40591-T62), rabbit polyclonal antibody against SARS-CoV-2 spike S2 (40590-T62), and rabbit monoclonal antibody against SARS-CoV/SARS-CoV-2 nucleocapsid (40143-R001). The goat anti-rabbit IgG-Fc antibody conjugated with HRP reporter (SSA003) served as the secondary antibody.

### 2.4. Plasmid DNA Transfection and RNA Extraction

Before transfection, 3.5 × 10^6^ HL-CZ cells were seeded into T25 flasks containing RPMI medium supplemented with 15% fetal bovine serum, and incubated at 37 °C in the presence of 5% CO_2_. At 22 h post-seeding, plasmid transfection was carried out. For each reaction, 7 µg of plasmid DNA was added to 14 µL of Lipofectamine 3000 (Life Technologies, Carlsbad, CA, USA), and serum-free RPMI medium was added to a final volume of 250 µL. After 5 min, 250 µL of mixture consisting of 14 µL of P3000 Enhancer reagent and serum-free RPMI were added to the diluted DNA. The DNA-lipid complex was further incubated for 30 min. After this, 500 µL of the DNA-lipid complex was added to the respective flask with HL-CZ cells. The transfected cells (1 million cells) were harvested after 24 h for RNA isolation using the RNeasy kit (Qiagen, Hilden, Germany) according to the manufacturer’s protocol. The purified RNA samples were kept at −80 °C for long-term storage. Each experimental condition comprised five biological replicates.

### 2.5. Protein Extraction, SDS-PAGE, and Western Blotting

Transfected HL-CZ cells (1 million cells) were harvested at 24 h post-transfection in RIPA buffer (Thermo Fisher Scientific, Waltham, MA, USA) supplemented with protease and phosphatase inhibitor (Pierce—one tablet per 10 mL of RIPA buffer). Protein quantification was performed using the BCA protein assay kit (Pierce; Thermo Fisher Scientific, Waltham, MA, USA), where 2 mg/mL BSA was used to construct the standard curve. Laemmli buffer (6×) was added to 30 µg of each purified protein lysate to a final concentration of 1×. The protein samples were incubated at 100 °C for 5 min, and then centrifuged at 10,000 rpm for 5 min. The purified protein lysates were loaded onto a 10% SDS-PAGE gel. The gels were electrophoresed at 60 V for 30 min, and subsequently at 100 V for 2 h. The proteins were then transferred onto an activated PVDF membrane in transfer buffer at 100 V for 1 h. The membrane was blocked in 5% milk prepared in 0.1% PBST for 1 h, followed by overnight incubation with primary antibody (1:1000 dilution) in 5% milk and 0.1% PBST at 4 °C. The blots were washed thrice in 0.1% PBST for 5 min each, and subsequently incubated with secondary antibody (1:3000 dilution) for 2 h. The blots were washed thrice in 0.1% PBST for 5 min each, and then incubated in WesternBright Enhanced Chemiluminiscent HRP substrate (Advansta, San Jose, CA, USA), prior to imaging using the ChemiDoc XRS imager (Bio-Rad, Hercules, CA, USA).

### 2.6. Confocal Microscopy

About 10,000 cells were concentrated onto each glass slide using Cytospin. The slides were then incubated in permeabilization buffer (0.5% Triton X-100 in Tris-buffered saline or TBS) for 30 min, and blocked in donkey serum blocking buffer (5% donkey serum, 1% BSA, 0.1% Triton X-100, 0.05% Tween-20 in TBS) for 2 h at room temperature. The primary antibodies used for immunofluorescence (1:50) were against spike S1, and S2. Each primary antibody was diluted in 1% BSA, and incubated overnight at 4 °C. The slides were then washed thrice using TBS with 0.05% Triton X-100 (TBST) for 3 min each, before incubation with TBST-diluted secondary antibody of donkey anti-rabbit Alexa Fluor 555 (1:200, A-31572, Thermo Scientific) at room temperature for 1 h. The slides were then washed thrice with TBST, and 15 mM 4′,6-diamidino-2-phenylindole (DAPI, diluted in TBS) was then added and incubated for 5 min, before washing thrice with TBST for 3 min each, followed by one final wash in TBS. Coverslips were mounted using ProLong Diamond Antifade Mountant (Thermo Scientific), and kept at 4 °C until fluorescence microscope imaging.

### 2.7. RNA-Sequencing

Extracted RNAs from three random samples in each of the transfected groups were subjected to mRNA sequencing (NovogeneAIT Genomics, Singapore). Briefly, after quality control procedures using Bioanalyzer, mRNA was enriched using oligo(dT) beads. The rRNA was removed using the Ribo-Zero kit. The mRNA was fragmented randomly by adding fragmentation buffer, and the cDNA was synthesized by using the mRNA template and random hexamers. Custom second-strand synthesis buffer (Illumina, San Diego, CA, USA), dNTPs, RNase H, and DNA polymerase I were added to initiate the second-strand synthesis. Following a series of terminal repair, A ligation, and sequencing adaptor ligation, the double-stranded cDNA library was completed through size selection and PCR enrichment. The libraries were processed at a sequencing depth of 40M reads per sample, and using paired-end 150 bp reads on an Illumina HiSeq instrument.

### 2.8. Data Processing

The quality of the raw data was first inspected using FASTQC, and the 5’ and 3’ adaptor sequences (5’-AATGATACGGCGACCACCGAGATCTACACTCTTTCCCTACACGACGCTCTTCCGATCT-3’ and 5’-GATCGGAAGAGCACACGTCTGAACTCCAGTCACATCACGATCTCGTATGCCGTCTTCTGCTTG-3’; where index sequences are six bases as underlined) were trimmed using Trimmomatic version 0.39 [18]. The processed reads were aligned against the GRCh38 *Homo sapiens* primary assembly (Ensembl release 102) using STAR version 2.7.6a [19] with default parameters. The reads mapping to the exons were quantified using featureCounts version 2.0.0 [20] with the following parameters: -t exon -g gene_id -p -B -C -F. Differential gene expression analysis was performed using the R package DESeq2 version 1.30.0 [21], after filtering genes with zero counts in all samples. The results are available in Supplementary Tables S2 and S3. For the identification of differentially expressed genes (DEGs), a Benjamini–Hochberg adjusted value of *p <* 0.05, and a minimum fold change value of 1.25 were considered. Volcano plots were generated using the R package EnhancedVolcano version 1.8.0 [22]. The normalized read counts of the identified DEGs were then used as input data for the online ClustVis tool at http://biit.cs.ut.ee/clustvis/ (accessed on 27 November 2020) to generate heatmaps with hierarchical clusters [23].

### 2.9. Gene Set Enrichment Analysis (GSEA)

The DESeq2-generated log2 fold change value of every gene in each comparison dataset was obtained for GSEA using the R package clusterProfiler version 3.18.0 [24], with org.Hs.eg.db being the source of annotation. Enriched gene ontology (GO) terms and Kyoto Encyclopedia of Genes and Genomes (KEGG) pathways with a Benjamini–Hochberg adjusted value of *p <* 0.05 were reported. In addition, to reduce the redundancy of enriched GO terms, the *simplify* function in clusterProfiler was used with a similarity threshold value of 0.7. For the visualization of significantly enriched KEGG pathways, the R package Pathview version 1.30.0 [25] was utilized.

### 2.10. Validation by Real-Time Reverse Transcription and Quantitative Polymerase Chain Reaction (RT-qPCR)

Extracted RNA was subjected to conventional reverse transcription (RT) for first-strand cDNA synthesis by mixing 0.5 μL of 500 ng/μL random hexamers (Promega, Madison, WI, USA), 1 μL of RNA (500 ng), and 2.25 μL of nuclease-free water to achieve a total volume of 3.75 μL. The mixture was then heated to 70 °C for 5 min before incubating on ice for 1 min. A total of 1.25 μL of MMLV reverse transcriptase 5× reaction buffer (Promega), 1.25 μL of dNTPs (10 μM), 0.16 μL of recombinant RNasin ribonuclease inhibitor (Promega), 0.25 μL of MMLV reverse transcriptase (Promega), and nuclease-free water were added to the mixture to give a final total reaction volume of 10 μL, and incubated at 37 °C for 1 h. Following first-strand synthesis, the cDNAs were diluted five times with nuclease-free water. Real-time PCR was then carried out for each sample using 5 μL of FastStart Essential DNA Green Master (Roche, Basel, Switzerland), 3 μL of nuclease-free water, 0.5 μL of target gene forward primer (10 μM), 0.5 μL of target gene reverse primer (10 μM) (Table 1), and 1 μL of diluted cDNA. Thermocycling was conducted using the following parameters: pre-incubation stage at 95 °C for 10 min, followed by 45 cycles each of denaturation (95 °C for 10 s), annealing (55 °C for 10 s), and elongation (72 °C for 10 s). Each sample was assayed as technical duplicates, and the fold change was calculated using the formula of 2^−ΔΔCT^.

## 3. Results

### 3.1. Successful Transfection of HL-CZ Cells

**Figure 1:**
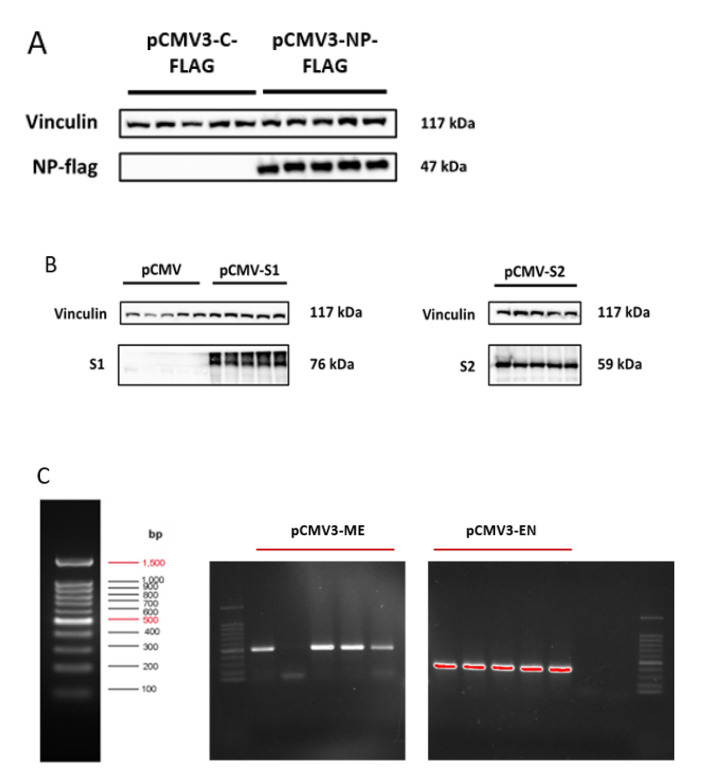
Confirmation of expression following plasmid transfection via Western blotting for (**A**) NP and (**B**) S1 and S2 proteins of SARS-CoV-2. (**C**) Direct PCR amplification followed by agarose gel electrophoresis exhibiting target amplicons for NSP16 (ME) and NSP15 (EN) transfections, which are absent in controls in the two lanes to the right of pCMV3-EN. The DNA size markers are shown in bp.

Successful transfection of HL-CZ cells was confirmed via Western blotting, confocal microscopy or PCR, depending on the availability of target antibody. As shown in Figure 1A,B, protein expression of NP, S1 and S2 was successfully detected in HL-CZ cells transfected with the respective plasmids. To confirm NSP15 and NSP16 plasmid transfection, direct PCR amplification was performed on whole cells using the corresponding primer sets (Table 1) after washing the cells with PBS thrice. Figure 1C shows successful transfection of NSP15 (EN) in all five replicates, with NSP16 (ME) transfection showing expression in four replicates. Representative confocal immunofluorescence images in Supplementary Figure S1A,B further verify spike S1 and S2 protein expression in the transfected HL-CZ cells which were stained orange with the corresponding S1 and S2 antibodies. Interestingly, both SARS-CoV-2 spike S1 and S2 exhibited predominant cytoplasmic localization similar to SARS-CoV spike expression in HL-CZ cells [15]. No S1 and S2 signal was observed in HL-CZ cells transfected with pCMV3 vector control and stained with S1 and S2 antibodies, as depicted in Supplementary Figure S1C,D.

### 3.2. RNA-Sequencing

In total, 390,479,125 and 543,230,844 paired-end reads were generated for the first set (PCMV, S1, and S2) and second set (PCMVF, NSP15, NSP16, NP) of samples, ranging from 40,327,168 to 51,967,126 reads per sample (Supplementary File S1). Following alignment of reads against the GRCh38 human reference genome, at least 90.4% of uniquely mapped reads in each sample were retained for subsequent differential expression analysis with DESeq2.

### 3.3. Identification of the Most Up-Regulated and Down-Regulated Genes

After normalization, DESeq2 was also utilized to screen the differentially expressed genes (DEGs) between HL-CZ cells transfected with SARS-CoV-2 genes versus cells transfected with empty PCMV or PCMVF vectors (controls). For spike S1-transfected HL-CZ cells, 82 DEGs were identified, among which 77 and five were significantly up-regulated and down-regulated, respectively. For spike S2-transfected cells, 42 DEGs were detected—27 up-regulated and 15 down-regulated (Supplementary File S2). For NSP15-transfected cells, 50 DEGs were detected—36 up-regulated and 14 down-regulated. For NSP16-transfected cells, 38 DEGs were identified, comprising 15 up-regulated and 23 down-regulated genes. For NP-transfected cells, only four up-regulated DEGs were found (Supplementary File S3). The above details are summarized in Table 2.

### 3.4. Hierarchical Clustering of DEGs

**Figure 2:**
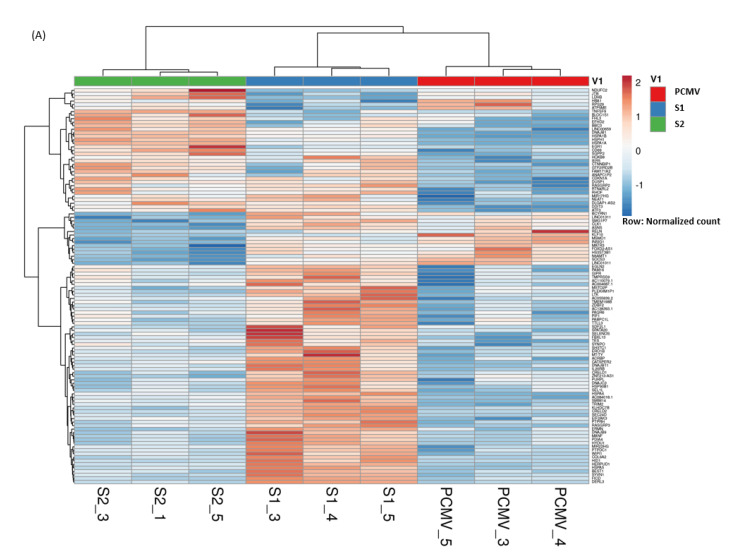

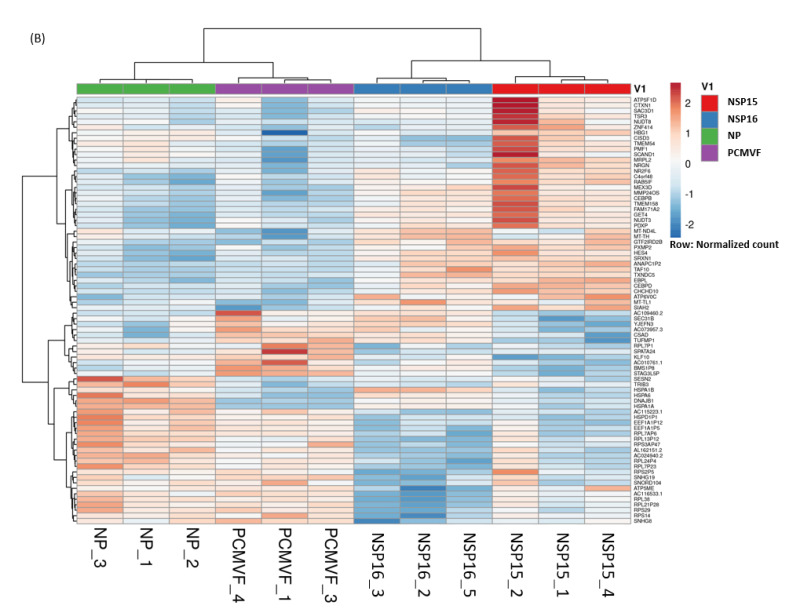
Heatmap across all the analyzed samples illustrating significant DEGs (log2 FC > 0.32, padj < 0.05) of HL-CZ cells transfected with (**A**) SARS-CoV-2 S1 and S2 versus PCMV vector control, and (**B**) SARS-CoV-2 NSP15, NSP16, NP versus PCMVF vector control.

To ensure that the identified DEGs were distinguished well between the cells transfected with empty vector controls and the five different SARS-CoV-2 ORFs, hierarchically clustered heatmaps were generated. Rows correspond to genes, and columns to samples. Rows are centered; unit variance scaling is applied to rows. Rows are clustered using Euclidean distance and Ward linkage. Columns are clustered using correlation distance and Ward linkage. In the heatmaps based on 112 DEGs (Figure 2A) and 78 DEGs (Figure 2B), each group of transfection samples was shown to cluster together, as expected. The up-regulated genes are depicted in brown, while the down-regulated genes are in blue.

### 3.5. Volcano Plots

**Figure 3:**
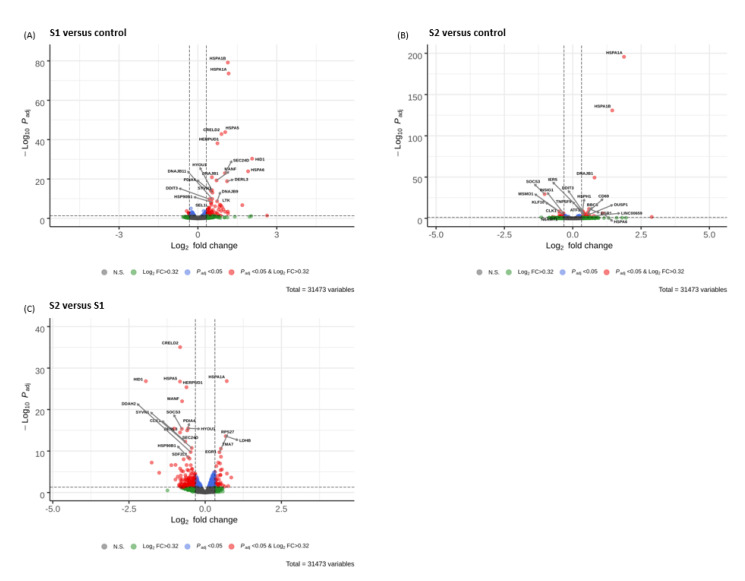

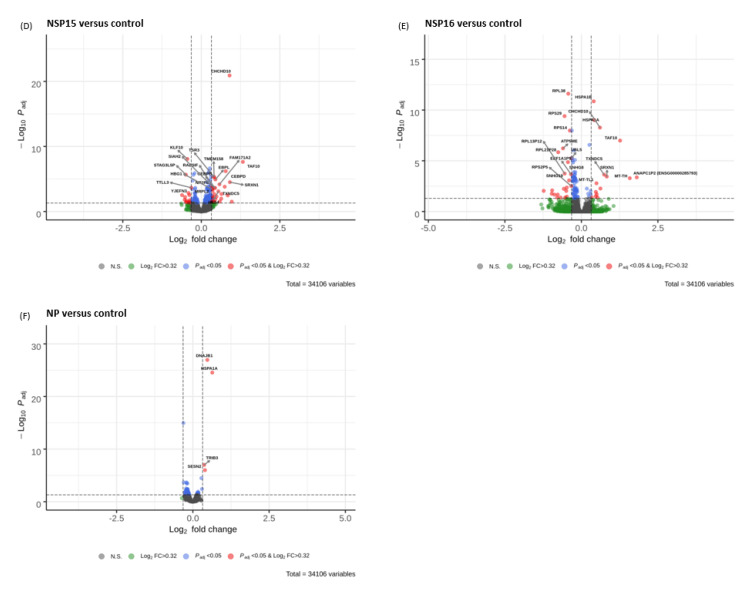

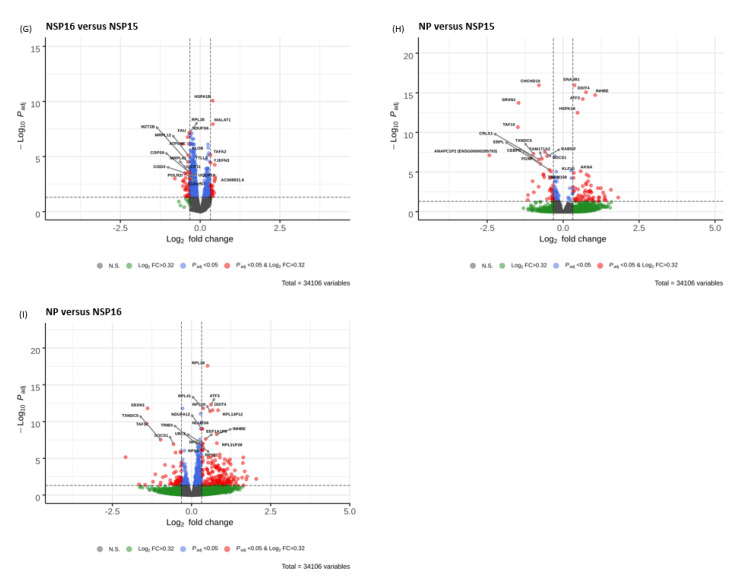
Volcano plots of the DEGs between transfections of (**A**) SARS-CoV-2 S1 versus PCMV vector control, (**B**) S2 versus PCMV, (**C**) S2 versus S1, (**D**) NSP15 versus PCMVF vector control, (**E**) NSP16 versus PCMVF, (**F**) NP versus PCMVF, (**G**) NSP16 versus NSP15, (**H**) NP versus NSP15, and (**I**) NP versus NSP16. Black dotted vertical lines highlight log_2_ fold changes of > 0.32, while black dotted horizontal lines represent padj < 0.05.

In Figure 3, the 20 most significant DEGs for each comparison group are shown in the volcano plots. The *y*-axis corresponds to the mean expression value of negative log10 (adjusted *p*-value), and the *x*-axis displays the log2 fold change (FC) value. The red dots represent the DEGs (padj < 0.05, log2 FC > 0.32) between gene-transfected and vector control cells. The blue dots represent the genes whose expression did not meet the set FC criteria (padj < 0.05, log2 FC ≤ 0.32), while green dots depict genes whose expression met the set FC criteria but were not statistically significant (padj ≥ 0.05, log2 FC > 0.32). The grey dots represent genes whose expression did not meet the set FC criteria, and were not statistically significant (padj ≥ 0.05, log2 FC < 0.32).

As illustrated in Figure 3A, HSPA1B, HSPA1A, HSPA5, CRELD2, and HERPUD1 were the five most significantly elevated genes in S1-transfected cells. Similarly, in the S2-transfected cells, HSPA1B and HSPA1A were found to be the most significantly elevated genes (Figure 3B). In NSP15-transfected cells, CHCHD10, TAF10, CEBPD, TMEM158, TSR3 and EBPL were the most significantly increased transcripts, while the most significantly down-regulated genes were KLF10 and HBG1 (Figure 3D). In NSP16-transfected cells, RPL38, RPS29, RPS14, ATP5ME, and RPL21P28 were the most significantly decreased genes, while HSPA1B, CHCHD10, HSPA1A, and TAF10 were the most significantly elevated (Figure 3E). In NP-transfected cells, DNAJB1, HSPA1A, SESN2, and TRIB3 exhibited the most significantly increased transcript levels (Figure 3F). Comparisons of volcano plots between cells transfected with S1, S2, NSP15, NSP16 and NP are also illustrated in Figure 3C,G,H,I.

While DNAJB1, HSPA1A, HSPA1B, and HSPA6 were all significantly up-regulated in HL-CZ cells transfected with spike S1 and S2 subunits, the expression of the former three genes were significantly higher in the S2-transfected cells compared to those transfected with the S1 subunit (Table 3). On the other hand, HSPA6 expression was significantly higher in the S1-expressing cells than in the S2-expressing cells.

### 3.6. Functional Enrichment Analysis

To identify enriched GO annotations and KEGG pathways associated with our differential expression analysis data, gene set enrichment analysis (GSEA) was performed using the clusterProfiler R package. The results are available in Supplementary Files S4–S8.

#### 3.6.1. Enriched GO Terms for Key Functional Categories

**Figure 4:**
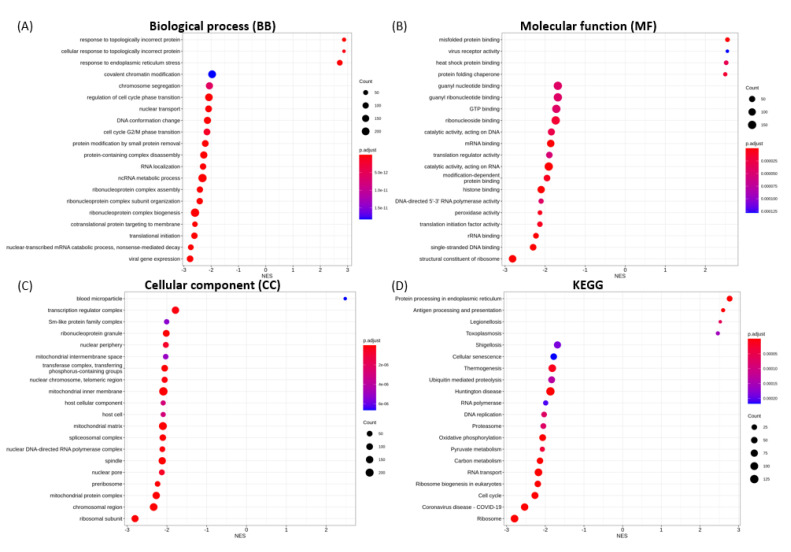
GSEA enrichment for SARS-CoV-2 spike S1 transfection illustrating the top 20 most significantly enriched terms for (**A**) BP, (**B**) MF, (**C**) CC and (**D**) KEGG pathways, in decreasing order based on normalized enrichment score (NES).

**Figure 5:**
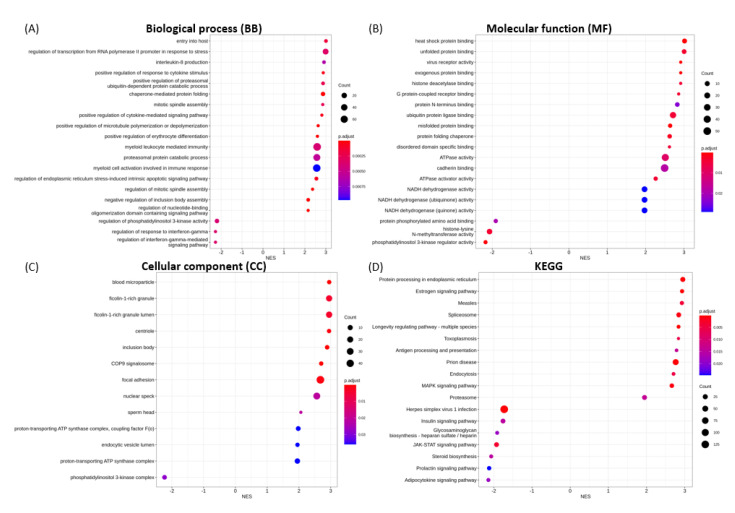
GSEA enrichment for SARS-CoV-2 spike S2 transfection illustrating the top 20 most significantly enriched terms for (**A**) BP, (**B**) MF, (**C**) CC and (**D**) KEGG pathways, in decreasing order based on normalized enrichment score (NES).

**Figure 6:**
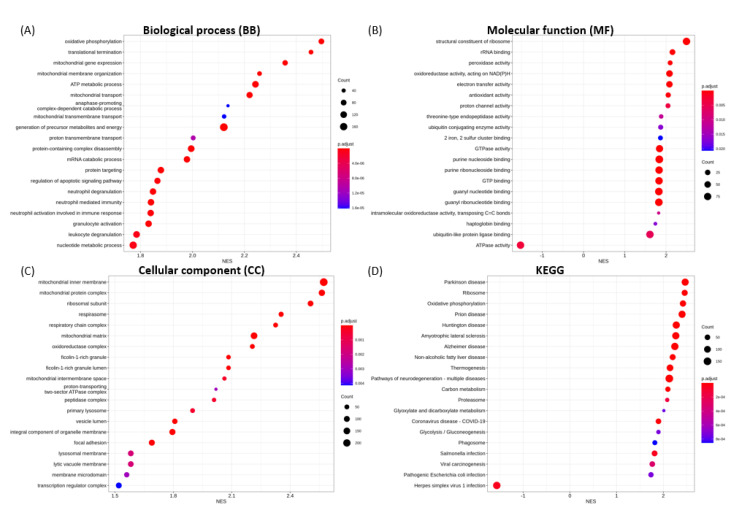
GSEA enrichment for SARS-CoV-2 NSP15 transfection illustrating the top 20 most significantly enriched terms for (**A**) BP, (**B**) MF, (**C**) CC and (**D**) KEGG pathways, in decreasing order based on normalized enrichment score (NES).

**Figure 7:**
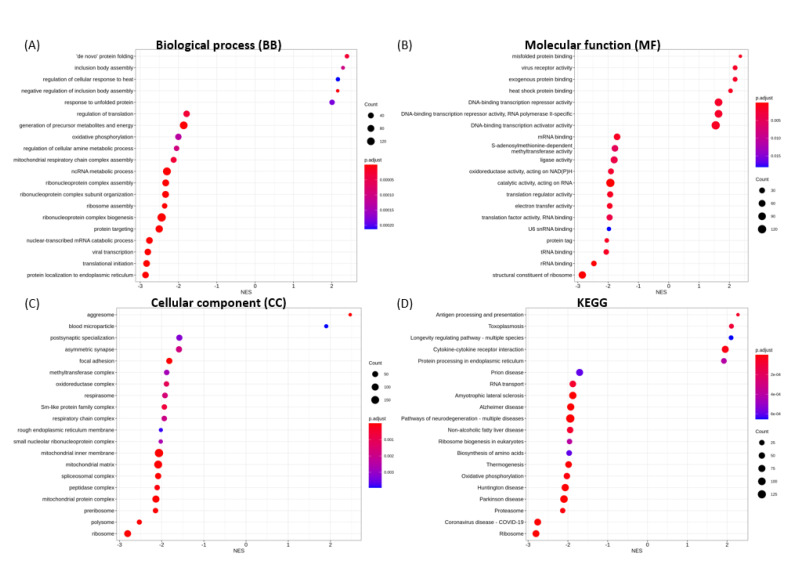
GSEA enrichment for SARS-CoV-2 NSP16 transfection illustrating the top 20 most significantly enriched terms for (**A**) BP, (**B**) MF, (**C**) CC and (**D**) KEGG pathways, in decreasing order based on normalized enrichment score (NES).

**Figure 8:**
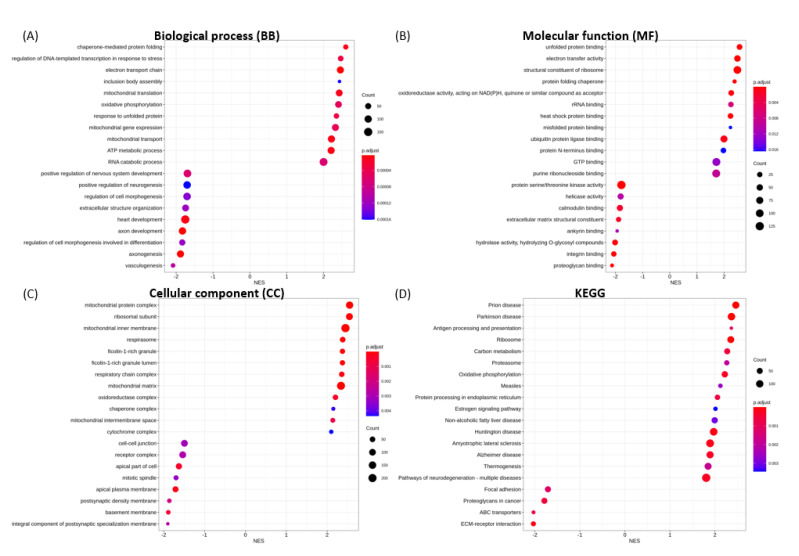
GSEA enrichment for SARS-CoV-2 NP transfection illustrating the top 20 most significantly enriched terms for (**A**) BP, (**B**) MF, (**C**) CC and (**D**) KEGG pathways, in decreasing order based on normalized enrichment score (NES).

The 20 most significantly enriched GO terms for biological process (BP), molecular function (MF), cellular component (CC), and KEGG categories in each transfection group are shown in Figure 4, Figure 5, Figure 6, Figure 7 and Figure 8.

The transcriptional changes in S1-transfected cells were shown to enrich for BP terms associated with topologically incorrect protein and endoplasmic reticulum (ER) stress (Figure 4A), MF terms including misfolded protein binding, virus receptor activity and heat shock protein binding (Figure 4B), and blood microparticle in the CC category (Figure 4C), in an up-regulated fashion. The most significantly enriched and down-regulated BP, MF and CC terms were: cell cycle G2/M phase transition, viral gene expression, ncRNA metabolic process, translational initiation (Figure 4A); structural constituent of ribosome, single-stranded DNA binding, rRNA binding (Figure 4B); and ribosomal subunit, chromosomal region, and mitochondrial protein complex (Figure 4C), respectively.

For spike S2, the enriched GO annotations for the most significantly up-regulated gene sets include entry into host, regulation of transcription from RNA polymerase II promoter in response to stress, interleukin-8 (IL-8) production (Figure 5A), heat shock protein binding, unfolded protein binding, virus receptor activity (Figure 5B), blood microparticle, ficolin-1-rich granule, and centriole (Figure 5C). The enrichment for down-regulated gene sets are the regulation of interferon-gamma-mediated signaling pathway, regulation of phosphatidylinositol 3-kinase (PI3-kinase) activity (Figure 5A), PI3-kinase regulator activity, histone-lysine N-methyltransferase activity, protein phosphorylated amino acid binding (Figure 5B), and PI3-kinase complex (Figure 5C). GSEA also indicated the up-regulation of enrichment gene sets associated with the positive regulation of the response to cytokine stimulus, positive regulation of the cytokine-mediated signaling pathway, myeloid leucocyte-mediated immunity, regulation of ER stress-induced intrinsic apoptotic signaling pathway, and regulation of nucleotide-binding oligomerization domain-containing signaling pathway (Figure 5A).

For NSP15 endoribonuclease, the enrichment for up-regulated gene sets include oxidative phosphorylation, translational termination, mitochondrial gene expression (Figure 6A), structural constituent of ribosome, rRNA binding, peroxidase activity (Figure 6B), mitochondrial inner membrane, ribosomal subunit, and respirasome (Figure 6C). The enrichment for down-regulated gene sets includes ATPase activity (Figure 6B). GSEA also demonstrated the up-regulation of enrichment gene sets associated with neutrophil degranulation, leukocyte degranulation, and neutrophil-mediated immunity (Figure 6A).

For NSP16 methyltransferase, the enrichment for up-regulated gene sets are “de novo” protein folding, inclusion body assembly, the regulation of cellular response to heat (Figure 7A), misfolded protein binding, viral receptor activity, heat shock protein binding (Figure 7B), aggresome, and blood microparticle (Figure 7C). The enrichment for down-regulated gene sets include protein localization to ER, translational initiation, viral transcription (Figure 7A), structural constituent of ribosome, rRNA binding, tRNA binding (Figure 7B), ribosome, polysome, and preribosome (Figure 7C). GSEA also revealed the down-regulation of enrichment gene sets associated with S-adenosylmethionine-dependent methyltransferase activity (Figure 7B).

For nucleocapsid protein (NP), the enrichment for up-regulated gene sets are chaperone-mediated protein folding, the regulation of DNA-templated transcription in response to stress, electron transport chain (Figure 8A), unfolded protein binding, electron transfer activity, the structural constituent of ribosome (Figure 8B), mitochondrial protein complex, ribosomal subunit, and mitochondrial inner membrane (Figure 8C). The enrichment for down-regulated gene sets include vasculogenesis, axonogenesis, the regulation of cell morphogenesis involved in differentiation (Figure 8A), proteoglycan binding, integrin binding, hydrolase activity (Figure 8B), an integral component of postsynaptic specialization membrane, basement membrane, and postsynaptic density membrane (Figure 8C). GSEA also showed down-regulation of enrichment gene sets related to axon development, positive regulation of neurogenesis, positive regulation of nervous system development, and cardiac development (Figure 8A).

#### 3.6.2. Enriched KEGG Pathways

**Figure 9:**
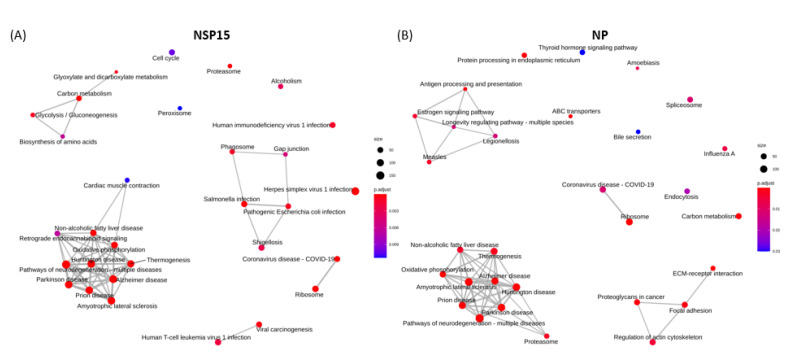
Network plot of enriched KEGG pathways for HL-CZ cell transfections of (**A**) NSP15 endoribonuclease and (**B**) NP nucleocapsid protein of SARS-CoV-2.

For S1-expressing cells, the most significantly up-regulated pathways were protein processing in ER, antigen processing and presentation, whilst ribosome, coronavirus disease, and cell cycle were the most significantly suppressed pathways (Figure 4D). For spike S2 protein, the most significantly enriched KEGG pathways comprised the activated protein processing for ER, and estrogen signaling pathways, as well as the down-regulated adipocytokine signaling, prolactin signaling, steroid biosynthesis, JAK-STAT signaling and insulin signaling pathways (Figure 5D). For NSP15 protein, the enriched up-regulated pathways were Parkinson disease, ribosome, and oxidative phosphorylation (Figure 6D). Interestingly, the gene sets involved in these pathways were also found to enrich for prion disease, Huntington disease, amyotrophic lateral sclerosis, Alzheimer disease, non-alcoholic liver disease, thermogenesis, and neurodegeneration (Figure 9A). For NSP16 protein, enriched up-regulated pathways were antigen processing and presentation, and cytokine-cytokine receptor interaction. The down-regulated pathways for NSP16 were ribosome and coronavirus disease (Figure 7D). For NP protein, prion disease and Parkinson disease pathways were among the most activated KEGG pathways. The down-regulated pathways with overlapping gene sets were ECM-receptor interaction, ABC transporters, proteoglycans in cancer, and focal adhesion (Figure 8D and Figure 9B).

### 3.7. Validation of Expression of Selected Genes via RT-qPCR

To validate the differential expression analysis data based on RNA-Seq, RT-qPCR was conducted on three genes (SOC3, HSPA1, and HSA6) whose expression levels were substantially altered with a log_2_ fold change > 1.0. The results of both RNA-Seq and RT-qPCR are tabulated in Table 4, where the expression levels of the selected genes were generally comparable between both methods.

## 4. Discussion

Coronavirus replicates in the cytoplasm, and its life cycle is closely associated with the ER. The virus hijacks the ER to process its structural and nonstructural proteins—viral activities thus exert a profound impact upon the ER function of host cells [26,27]. During coronavirus replication, substantial amounts of heavily modified transmembrane proteins, including spike protein, are generated. The accumulation of nascent and unfolded viral proteins in the ER lumen may rapidly overload the folding capacity of the ER, thereby perturbing normal cellular functions and causing ER stress [28]. ER stress activates multiple cell-signaling pathways, collectively termed the unfolded protein response (UPR), to regulate gene expression to adjust the biosynthetic burden and capacity of the ER to maintain homeostasis [29]. When ER is severely damaged, the UPR triggers apoptosis [30,31]. Interestingly, the related SARS coronavirus (SARS-CoV) spike protein can sufficiently induce transcriptional activation of UPR effectors [28]. Transcriptomics data identified activating transcription factor 3 (ATF3), DNA damage-inducible transcript 3 (DDIT3), and dual-specificity phosphatase 1 (DUSP1) to be induced by SARS-CoV, Middle East respiratory syndrome coronavirus (MERS-CoV), and human coronavirus (HCoV) [32,33,34]. We observed that ATF3 (an UPR-regulated transcription factor) and DDIT3 (a pro-apoptotic transcription factor) were also induced by both SARS-CoV-2 spike S1 and S2 subunits (Table 3). ATF3 is a hub of the cellular adaptive-response network that connects multiple extracellular signals, such as ER stress, cytokines, chemokines, and lipopolysaccharide. ATF3 plays vital roles in modulating metabolism, immunity, and oncogenesis [35]. DDIT3 plays important roles in ER stress-induced apoptosis and autophagy. In addition, bovine viral diarrhea virus infection induces high DDIT3 expression, which targets mitochondrial antiviral signaling (MAVS), leading to the inhibition of type I interferon and interferon-stimulated gene production, thereby promoting viral replication [36]. Although DUSP1 does not inhibit the antiviral response, it negatively regulates virus-induced JNK/p38 MAPK phosphorylation, and DUSP1 is upregulated before being subjected to proteasomal degradation. Interaction with the JNK-interacting protein 1 scaffold protein prevents dephosphorylation of JNK by DUSP1, likely explaining that AP-1 activation and downstream cytokine production are protected from DUSP1 inhibition. Importantly, DUSP1 promotes virus-induced apoptosis and suppresses cell migration in infected cells [37], hence suggesting a possible role of DUSP1 in the regulation of tissue damage and repair during infection by SARS-CoV-2.

Interestingly, SARS-CoV-2 S1 expression downregulated biological processes associated with DNA conformational change, cell cycle, and nuclear transport. These findings are congruent with in vitro and in vivo transcriptomic analyses, showing that SARS-CoV-2 generates a unique gene signature enriched for cell death as well as leukocyte activation [38]. It would be interesting to study whether SARS-CoV-2 S1 may be linked to immunosenescence or diminished immunologic competence, especially in elderly individuals [39].

Akin to a double-edged sword, UPR may either be beneficial or detrimental to the virus. To survive ER stress, viruses have developed different strategies to modulate the UPR. HSPA1A, HSPA1B and HSPA6 are members of the heat shock protein 70 (HSP70) family, while DNAJB1 is a member of the HSP40 family. HSP70 and HSP40 co-operate to prevent aggregation of misfolded proteins [40]. Heat shock proteins are documented to protect against ER stress-induced apoptosis [41,42]. Expression of HL-CZ cells with SARS-CoV-2 spike S1 and S2 proteins were found to significantly up-regulate these HSP family members (Table 4). Thus, the up-regulation of these heat shock proteins may represent the host’s response to counter ER stress-induced apoptosis arising from viral infection. Intriguingly, intracellular HSP70 exerts anti-inflammatory effects, but this protein can be released as extracellular HSP70, which activates pro-inflammatory pathways instead [43]. Another heat shock protein, HSP90β interacts with and stabilizes NP of MERS-CoV, and inhibition of HSP90 by RNA interference or by 17-AAG significantly suppresses MERS-CoV replication [44]. It is also noteworthy that HSPA1A and HSP90 are also significantly upregulated during SARS-CoV infection of Vero E6 cells [45].

In this study, our data demonstrated that interferon-gamma-mediated signaling pathway, PI3-kinase regulator activity, and JAK-STAT signaling pathway were all down-regulated in the S2-expressing pro-monocytic cells (Figure 5A,D). Type 2 interferon-gamma stimulation activates PI3-kinase and its effector kinase AKT, triggering JAK-STAT signaling and gene expression [46]. This suggests that SARS-CoV-2 spike S2 subunit may possess immunomodulatory functions by blocking interferon signaling compared to its S1 counterpart, and that suppression of interferon by S2 may be crucial for viral replication. Interferon immunity plays an essential role in controlling SARS-CoV-2 infection [47]. SOCS3 is a potent and specific inhibitor of IL-6 signaling, and SOCS3 binds to JAKs to inhibit JAK activity [48]. RT-qPCR revealed significantly reduced SOCS3 mRNA in spike S2-expressing cells, implying enhanced IL-6 signaling which is a key part of the cytokine storm occurring in severe COVID-19. An aberrant STAT pathway is also proposed to be central to COVID-19 pathophysiology [49]. In addition, it has been documented that human macrophages can be activated by spike protein of the related SARS-CoV to induce TNF-alpha, IL-6 and IL-8 [50].

Furthermore, IL-8 gene production was up-regulated with S2 expression (Figure 5A), while cytokine-cytokine receptor interaction was up-regulated with NSP16 expression (Figure 7D). IL-8 is a potent pro-inflammatory cytokine that mediates the recruitment and activation of neutrophils during inflammation, and IL-8 may contribute to COVID-19 pathology. Indeed, excessive neutrophils and neutrophil extracellular traps are present in the lungs of critically ill COVID-19 patients [51], and they act as critical drivers of progressive pulmonary impairment [52]. Interestingly, we previously showed the upregulated expression of IL-8 receptor A (IL8RA) in Vero E6 cells infected with SARS-CoV [45], as well as sharp up-regulation of IL-8 receptor transcript β in HL-CZ cells expressing spike protein of SARS-CoV [15].

SARS-CoV-2 replicates more efficiently than SARS-CoV in human lung tissues, and can upregulate critical inflammatory mediators [53]. RNA-Seq transcriptomic profiling analyses of airway and/or blood samples from severe COVID-19 patients reveal the predominance of aberrant monocytes with heightened levels of myeloid chemoattractants in airways [54]. SARS-CoV-2 infection of human nasal epithelial cells can significantly induce CXCL10 cytokine production [55]. Moreover, SARS-CoV-2 infects alveolar macrophages which produce T-cell chemoattractants, which in turn induce inflammatory cytokine release by macrophages to promote T-cell activation, thus constituting a positive feedback loop that drives alveolar inflammation [56]. Hence, SARS-CoV-2-infected macrophages contribute to viral spread, exuberant inflammation, and activation-induced lymphocytic cell death [57].

The KEGG adipocytokine signaling pathway describes signaling cascades arising from adipocytokines that are implicated in insulin resistance and sensitivity. In this study, dysfunctional profile of adipokines, which links various metabolic diseases (such as insulin resistance) to inflammatory manifestations, was associated with S2 expression (Figure 5D). Thus, aberration of the adipocytokine signaling pathway caused by the spike S2 subunit of SARS-CoV-2 may be linked to patients with underlying metabolic disorders, such as type 2 diabetes and obesity, who are at higher risk of developing severe complications of COVID-19 [58]. Adipocytokine dysfunction may lead to a specific immune environment that predisposes diabetic and obese patients to respiratory failure [59]. Krause et al. [60] also hypothesized that the heat shock response may be a determinant of complications in COVID-19 patients with co-morbidities of diabetes and obesity. Understanding the multiple and interrelated factors linking SARS-CoV-2 infection, adipocytokine signaling, global inflammation, and metabolic disorders (e.g., type 2 diabetes and obesity) can provide better insights into the interplay of these conditions and physiological states.

Enriched down-regulated pathways for NP include extracellular matrix (ECM)-receptor interaction and focal adhesion (Figure 8D and Figure 9B). This is consistent with a previous report that proteins associated with focal adhesion and interactions with the ECM receptors were all decreased in fresh lung tissues obtained from newly deceased patients with COVID-19 pneumonia [61]. This suggests that SARS-CoV-2 infection may lead to dysregulation of the extracellular microenvironment in the lung, revealing a potential mechanism of virus-related lung injury in severe COVID-19. During infection, it is estimated that 10-fold more NP protein may be produced in comparison with spike protein [62]. In COVID-19, it is also noteworthy that antibody responses to NP are elevated in deceased patients, whereas spike-specific antibody responses are enriched among convalescent patients [63]. In addition, SARS-CoV-2 NP is proposed to interact with 15 human proteins, mainly associated with RNA processing or stress granule regulation [64]. We previously reported that SARS-CoV NP can induce actin reorganization and apoptosis in cells in the absence of growth factors [65]. SARS-CoV NP also interacts with host cyclin-CDK complex to regulate the cell cycle [66], and it binds to translation elongation factor 1 alpha to inhibit proliferation of cells including lymphocytes [67].

Thioredoxin domain containing 5 (TXNDC5) was significantly up-regulated in cells expressing NSP15 (EN) and NSP16 (ME) proteins (Table 3). This ER-resident protein is a critical mediator of cardiac and renal fibrosis [68]. SARS-CoV-2 NSP15 also functions as a potent interferon antagonist [69]. Coronavirus NSP16 interacts with NSP10 to form a stable complex to mediate 2’-O-methyltransferase activity [70].

A recent study of SARS-CoV-2 infection on the host proteome indicated the perturbation of several pathways associated with neurodegeneration, including but not limited to Parkinson and Huntington diseases [71]. Similarly, in our study, we showed the up-regulation of enriched genes associated with the perturbation of pathways of neurodegeneration in cells expressing NSP15 and NP (Figure 6D, Figure 8D, Figure 9A and Figure 9B). GSEA on gene expression data from tissues donated by idiopathic Parkinson disease (iPD) patients suggest the potential contribution of human coronaviruses in the pathogenesis of iPD [72]. In a case report, a patient whose first manifestations of Creutzfeldt–Jakob disease occurred in tandem with symptomatic onset of COVID-19 led the authors to hypothesize that the cascade of systemic inflammatory mediators in response to the virus accelerated the pathogenesis of his prion disease [73]. Given the neuroinvasive potential of SARS-CoV-2, deeper investigation is warranted into the virus’ contribution to the long-term development of neurodegenerative disease.

In contrast to live SARS-CoV-2 infection, this study elucidated the host transcriptomic profiles of critical SARS-CoV-2 proteins when expressed individually in human pro-monocytic cells, thus dissecting their effects and providing useful insights into their specific roles during viral infection. Such studies can enhance our understanding of the “infectomics” of viral infections in different human cell types, including monocytes and macrophages. To follow up on the RNA-Seq data, future detailed analyses of selected differently expressed genes and proteins of interest may be conducted by real-time RT-qPCR, Western blotting and other in vitro techniques, and by harnessing gene-knockout and other animal models to further investigate their specific functions in vivo during SARS-CoV-2 infection. Mice expressing human ACE2 and challenged with recombinant SARS-CoV-2 spike protein can elucidate the latter’s contribution to COVID-19 pathology [74]. Another strategy for transcriptomic and other analyses is to infect cells expressing human ACE2 receptor with lentiviral particles expressing SARS-CoV-2 proteins, e.g., spike glycoprotein [75]. Instead of expressing one viral protein at a time, combined expression of viral proteins may also yield useful insights into their synergistic roles [76].

## Figures and Tables

**Table 1:** Sequences of human gene primers for RT-qPCR validation or classical PCR amplification.

Gene	Forward Primer (5’–3’)	Reverse Primer (5’–3’)
GAPDH (reference)	ACCCACTCCTCCACCTTTGAC	TCCACCACCCTGTTGCTGTAG
SOCS3	CGCCTCAAGACCTTCAGCTC	TGAAGAAGTGGCGCTGGTC
HSPA1	GAGCGCAACGTGCTCATCT	ACCTCGAAGATGCCGTCGT
HSPA6	TGGCTGCCAAAAACTCGCTG	CAGGCAAGGACTTCCCGACA
NSP15	TTGTGCACCACTCACTGTCT	GAACCTGTTTGCGCATCTGTT
NSP16	AAGACAGTGGTTGCCTACGG	TCGCGTGGTTTGCCAAGATA

**Table 2:** Summary of the numbers of up-regulated and down-regulated DEGs in HL-CZ cells transfected with SARS-CoV-2 spike S1, S2, NSP15, NSP16, and NP plasmids.

Transfection Group	Up-Regulated DEGs	Down-Regulated DEGs	Total Number
S1	77	5	82
S2	27	15	42
NSP15	36	14	50
NSP16	15	23	38
NP	4	0	4

**Table 3:** Average log_2_ fold change of selected human genes of interest across RNA samples of HL-CZ cells expressing different SARS-CoV-2 genes (versus plasmid vector controls).

	Log_2_ Fold Change (Adjusted *p*-Value)								
Gene	S1 vs. PCMV	S2 vs. PCMV	NSP15 vs. PCMVF	NSP16 vs. PCMVF	NP vs. PCMVF	S1 vs. S2	NSP16 vs. NSP15	NP vs. NSP15	NP vs. NSP15
**ATF3**	0.41 (1.45 × 10^−5^)	0.43 (5.20 × 10^−6^)	NS	NS	NS	NS	NS	0.65 (6.10 × 10^−15^)	0.61 (4.90 × 10^−13^)
**CHCHD10**	NS	NS	0.90 (1.22 × 10^−21^)	0.61 (5.36 × 10^−9^)	NS	NS	−0.29 (0.014)	−0.80 (1.10 × 10^−16^)	−0.50 (1.63 × 10^−6^)
**DDIT3**	0.44 (1.75 × 10^−10^)	0.39 (6.53 × 10^−8^)	NS	NS	NS	NS	NS	NS	NS
**DNAJB1**	0.53 (1.18 × 10^−21^)	0.80 (4.30 × 10^−50^)	NS	0.26 (2.71 × 10^−7^)	0.47 (1.12 × 10^−27^)	0.26 (4.97 × 10^−5^)	0.16 (0.0027)	0.37 (1.10 × 10^−16^)	0.21 (2.40 × 10^−5^)
**DUSP1**	0.57 (5.21 × 10^−5^)	0.60 (2.84 × 10^−5^)	NS	NS	NS	NS	NS	NS	0.32 (0.026)
**HSPA1A**	1.67 (2.48 × 10^−74^)	1.88 (2.10 × 10^−196^)	NS	0.42 (9.64 × 10^−10^)	0.44 (1.75 × 10^−10^)	0.71 (1.36 × 10^−27^)	0.26 (0.0006)	0.47 (3.28 × 10^−13^)	0.22 (0.0075)
**HSPA1B**	1.14 (6.91 × 10^−80^)	1.44 (1.76 × 10^−131^)	NS	0.40 (1.38 × 10^−11^)	0.63 (2.88 × 10^−25^)	0.31 (1.17 × 10^−5^)	0.39 (8.66 × 10^−11^)	0.27 (5.84 × 10^−5^)	NS
**HSPA6**	1.91 (1.36 × 10^−24^)	1.11 (1.16 × 10^−6^)	NS	0.53 (0.043)	NS	−0.80 (7.75 × 10^−5^)	NS	0.54 (0.019)	NS
**IER5**	0.42 (6.31 × 10^−6^)	0.44 (5.20 × 10^−6^)	NS	NS	NS	NS	NS	NS	NS
**KLF10**	−0.28 (0.0013)	−0.37 (2.24 × 10^−6^)	−0.44 (8.37 × 10^−9^)	−0.23 (0.032)	NS	NS	0.21 (0.015)	0.35 (1.26 × 10^−5^)	NS
**TXNDC5**	NS	NS	0.15 (0.0002)	0.74 (0.0002)	NS	NS	NS	−0.98 (5.05 × 10^−8^)	−0.98 (2.91 × 10^−8^)

NS: Not statistically significant, *p* ≥ 0.05.

**Table 4:** Average log_2_ fold change of selected human genes of interest by RNA-Seq and real-time RT-qPCR of HL-CZ cells expressing SARS-CoV-2 spike S1 and S2 subunits.

		RNA-Seq	Real-Time RT-qPCR
Gene	Sample	Log_2_ Fold Change (*p*-Value)	Log_2_ Fold Change (*p*-Value)
SOCS3	S1	−0.261 (*p* ≥ 0.05)	−0.263 (*p* ≥ 0.05)
	S2	−1.028 (*p* < 0.001) *	−1.379 (*p* < 0.001) *
HSPA1A	S1	1.168 (*p* < 0.001) *	-
	S2	1.876 (*p* < 0.001) *	-
HSPA1B	S1	1.139 (*p* < 0.001) *	-
	S2	1.446 (*p* < 0.001) *	-
HSPA1	S1	-	1.356 (*p* = 0.001) *
	S2	-	1.367 (*p* = 0.004) *
HSPA6	S1	1.909 (*p* < 0.001) *	1.1506 (*p* = 0.035) *
	S2	1.109 (*p* < 0.001) *	0.379 (*p* ≥ 0.05)

* *p*-value < 0.05 is considered statistically significant. The HSPA1 PCR primers amplify both HSPA1A and HSPA1B genes.

## Data Availability

The RNA-Seq data presented in this study are publicly available in the GEO database under the series accession number GSE171080.

## Supplementary Materials

The following are available online at https://www.mdpi.com/article/10.3390/microorganisms9061193/s1: Supplementary Figure S1. Representative confocal immunofluorescence images of transfected HL-CZ cells. SARS-CoV-2 spike (A) S1 and (B) S2 protein expression was verified by orange staining of transfected HL-CZ cells using the corresponding S1 and S2 antibodies, respectively. Both SARS-CoV-2 spike S1 and S2 proteins exhibited predominant cytoplasmic localization in HL-CZ cells. No S1 and S2 signal was observed in HL-CZ cells transfected with pCMV3 vector control and stained with (C) S1 antibody and (D) S2 antibody. Supplementary File S1: Statistics for the quality of RNA sequencing data. Supplementary File S2: Differentially expressed genes for HL-CZ cell transfections with PCMV vector, SARS-CoV-2 spike S1 and S2 subunits. Supplementary File S3: Differentially expressed genes for HL-CZ cell transfections with PCMVF vector, SARS-CoV-2 NSP15 (EN), NSP16 (ME), and nucleocapsid protein (NP). Supplementary File S4: GSEA analysis for HL-CZ cell transfection with SARS-CoV-2 spike S1. Supplementary File S5: GSEA analysis for HL-CZ cell transfection with SARS-CoV-2 spike S2. Supplementary File S6: GSEA analysis for HL-CZ cell transfection with SARS-CoV-2 NSP15 endoribonuclease (EN). Supplementary File S7: GSEA analysis for HL-CZ cell transfection with SARS-CoV-2 NSP16 methyltransferase (ME). Supplementary File S8: GSEA analysis for HL-CZ cell transfection with SARS-CoV-2 nucleocapsid protein (NP).
