# Supplementary figures and images for: Comparative Transcriptomic and Molecular Pathway Analyses of HL-CZ Human Pro-Monocytic Cells Expressing SARS-CoV-2 Spike S1, S2, NP, NSP15 and NSP16 Genes
Source: Microorganisms. 2021 May 31;9(6):1193. doi: 10.3390/microorganisms9061193 (PMC8228226; doi:10.3390/microorganisms9061193)

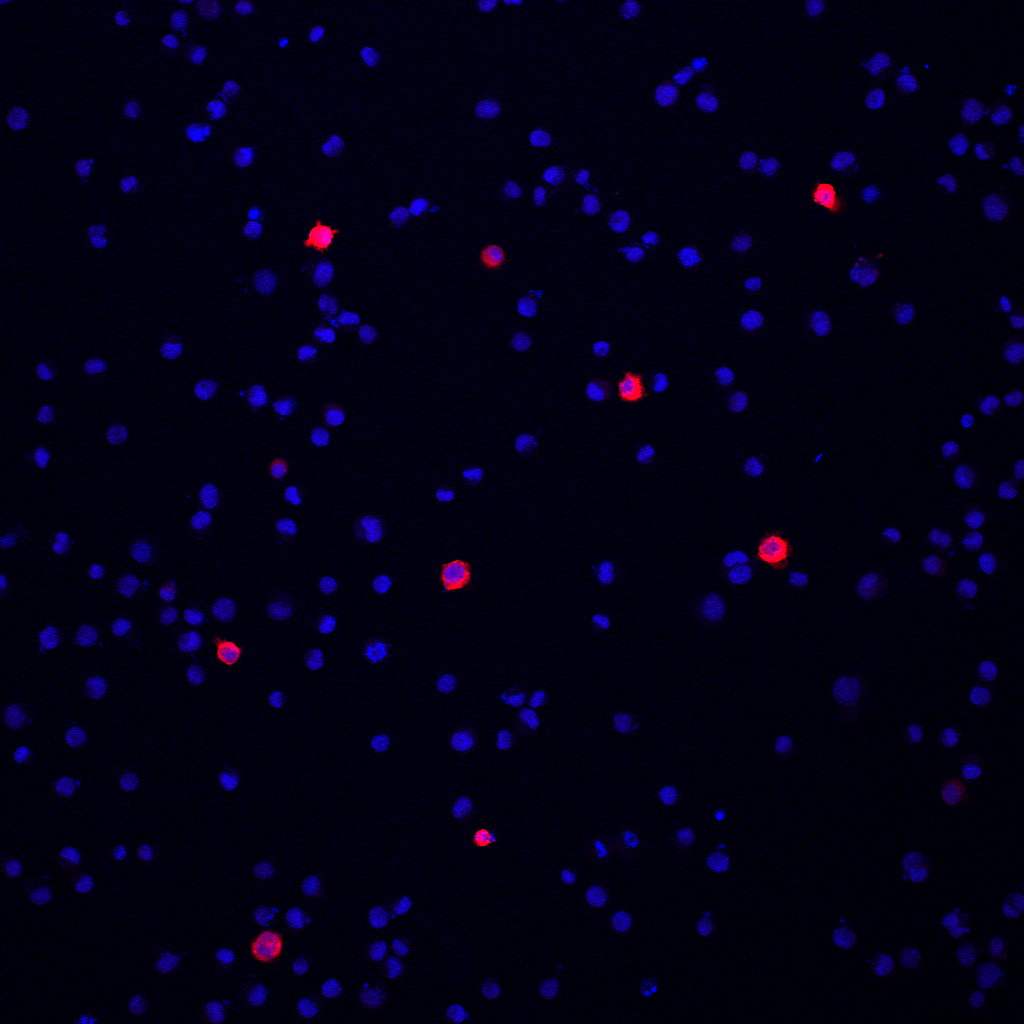

Supplement: Supplementary file 1 [file microorganisms-09-01193-s001.zip › Microorg Supp Material 30May 2021/Supplemenatry Figure 1B.tif]

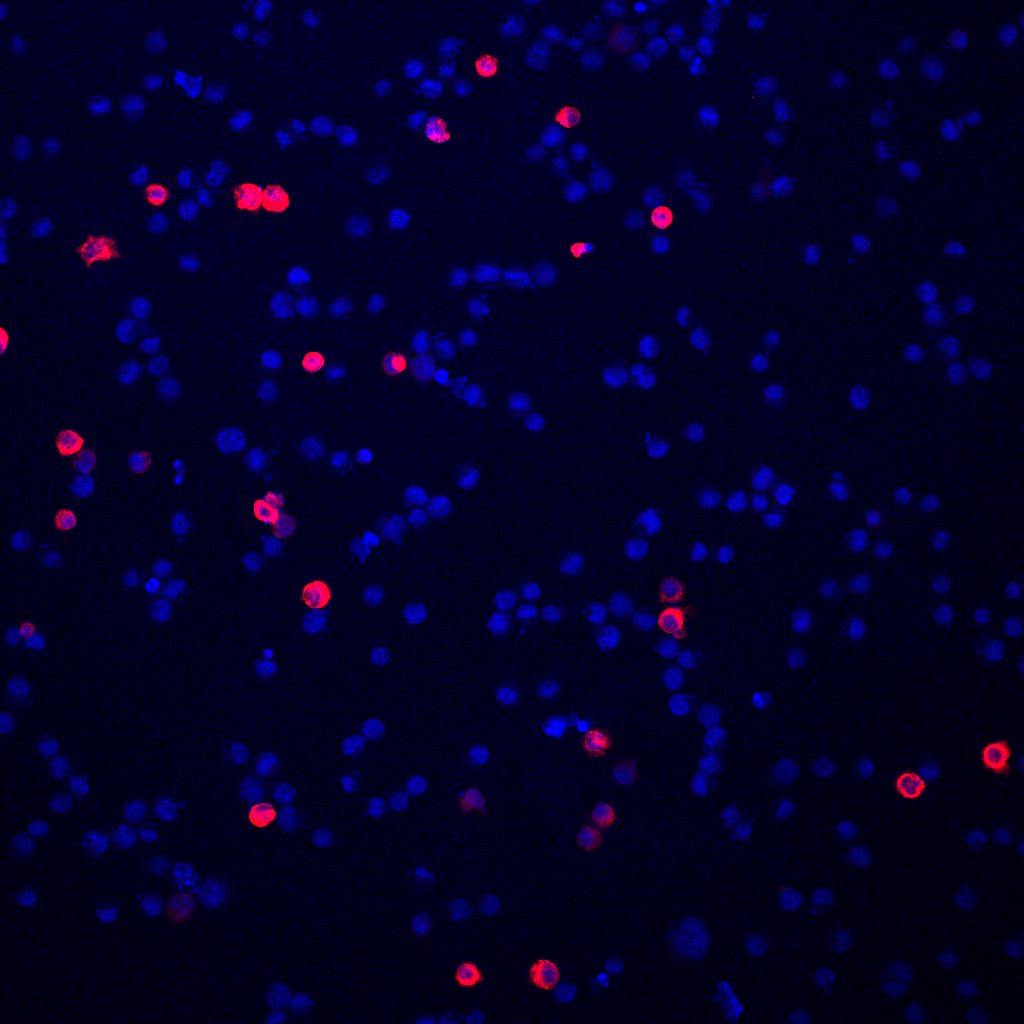

Supplement: Supplementary file 1 [file microorganisms-09-01193-s001.zip › Microorg Supp Material 30May 2021/Supplementary Figure 1A.tif]

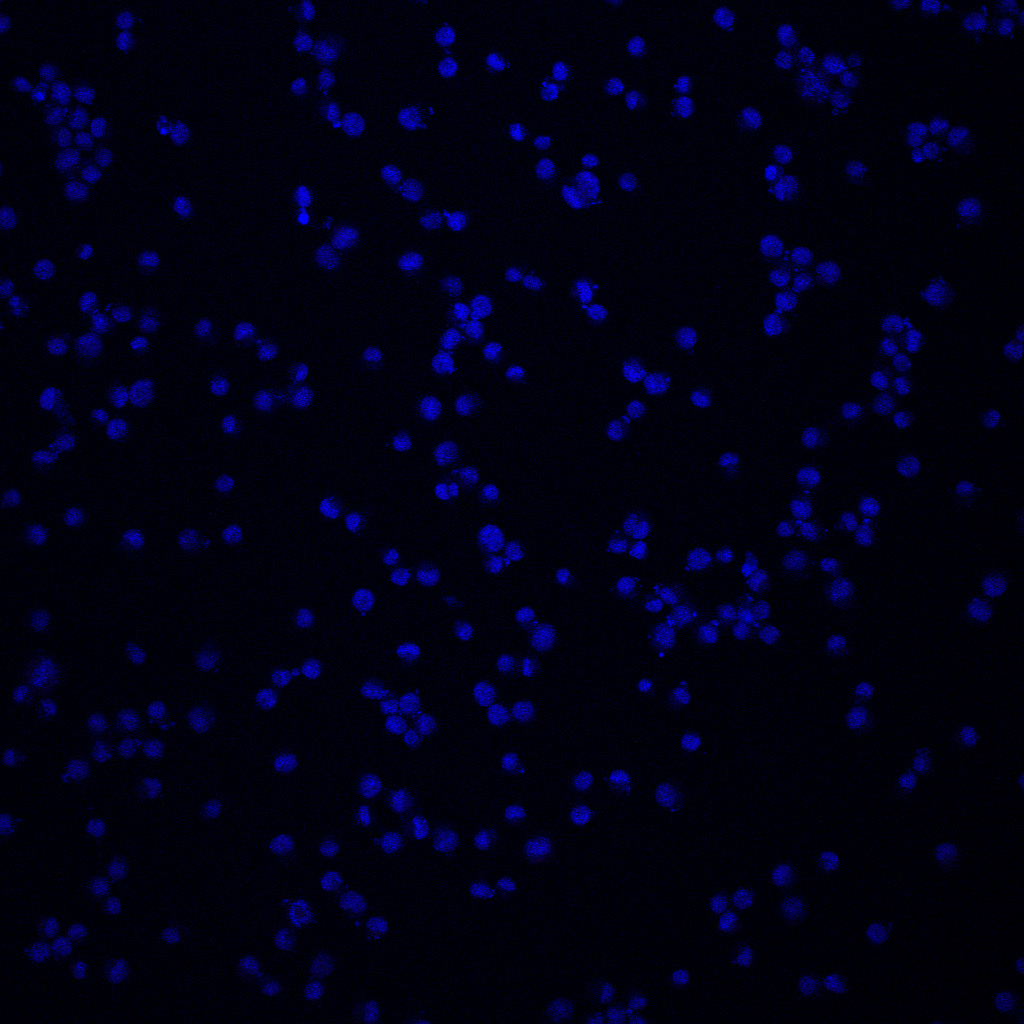

Supplement: Supplementary file 1 [file microorganisms-09-01193-s001.zip › Microorg Supp Material 30May 2021/Supplementary Figure 1C.tif]

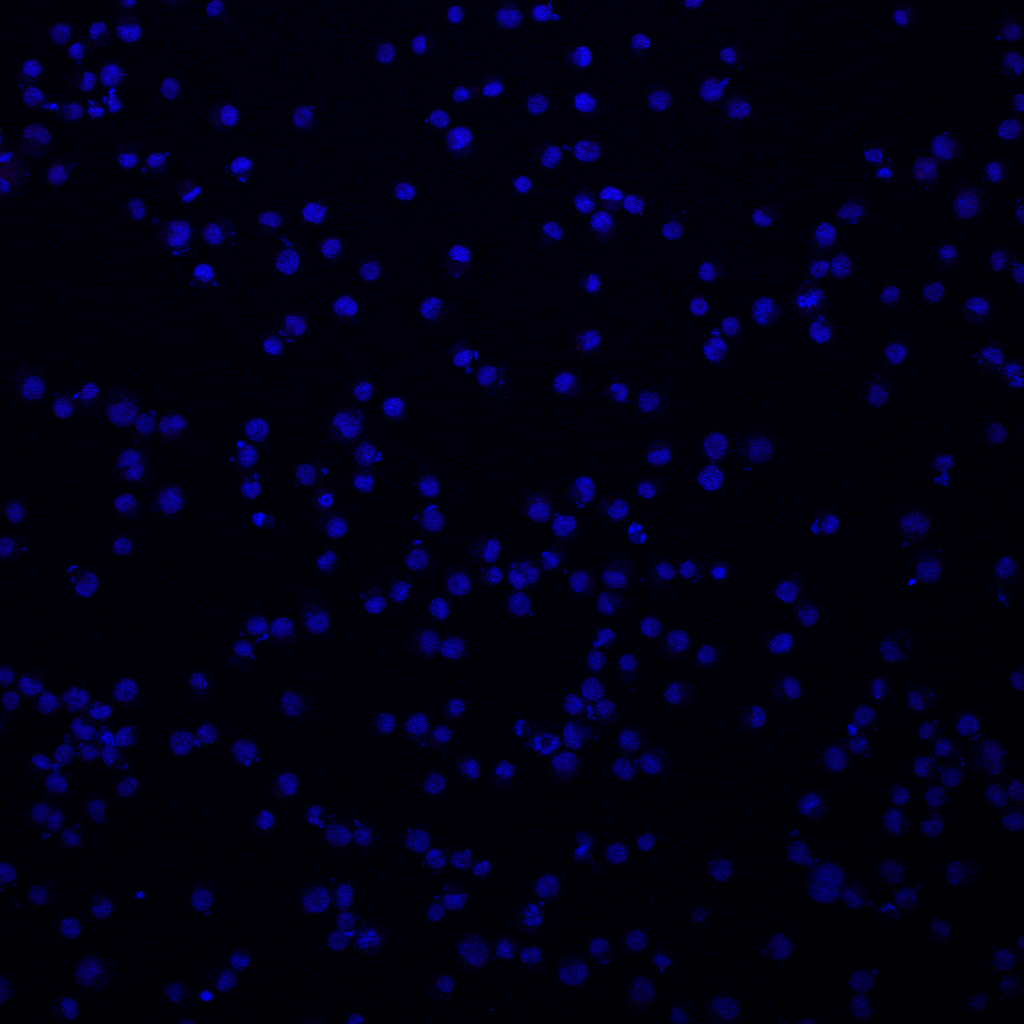

Supplement: Supplementary file 1 [file microorganisms-09-01193-s001.zip › Microorg Supp Material 30May 2021/Supplementary Figure 1D.tif]
